# Psychosis as a potential mental health consequence of racism

**DOI:** 10.1192/j.eurpsy.2023.1928

**Published:** 2023-07-19

**Authors:** F. B. Lazaridou, A. Heinz

**Affiliations:** 1 Department of Psychiatry and Psychotherapy, Charitè University of Medicine Berlin; 2 National Discrimination and Racism Monitor, German Institute for Integration and Migration Research; 3 Department of Migration, Mental and Physical Health and Health Promotion, Berlin Institute of Integration and Migration Research; 4 Department of Psychiatry and Psychotherapy, Alexianer St. Hedwig-Hospital, Berlin, Germany

## Abstract

**Introduction:**

Evidence shows that racism can have a negative effect on mental health in the lived experiences of Black people and People of Colour. In critical theory discourse including postcolonial and decolonial approaches, racism is suggested to be an everyday phenomenon. Additionally, racism specifically targets the perceived cultural and phenotypic foreignness of Black migrants and migrants Of Colour, as well as the ascribed migrant status attributed to the perceived foreignness of racialized persons who do not actually have any direct migration experiences.

**Objectives:**

The stigma associated with severe mental disorders such as psychosis has historically been applied to Black people and People of Colour who have been engaged in anti-racist activism as a form of punishment and social control. Higher incidence rates of psychosis in racialized communities have frequently been conceptualized as cultural differences in family composition and levels of expressed emotion in families. The objective of this study is to sensitively investigate psychosis as a potential mental health consequence of racism.

**Methods:**

The incidence rates of psychosis - positive symptoms, negative symptoms, non-affective psychosis disorders and first episode psychosis - among migrants by country of migration were compiled in an umbrella review, which offers a summary of meta-analyses. Quantitative research has the limitation of enabling the observation of patterns but not allowing an understanding of the reasons behind them to be theorized through the data. Therefore, qualitative methods complement the quantitative data. Twenty people of diverse genders who self-identified as Black people or People of Colour in Berlin were interviewed about their experiences of racism and sexism and about how those experiences affected their mental health.

**Results:**

The umbrella review found an association between migration and psychosis, with migration from the Caribbean and African countries showing the strongest correlation. A constant comparative analysis of the qualitative data suggests that racism contributes to the emergence of a subclinical psychosis symptomatology profile that consists of a sense of differentness, negative self-awareness, paranoid ideation regarding general persecution, and self-questioning with self-esteem instability.

**Conclusions:**

The findings are interpreted as a situational diagnosis, as coined by the psychiatrist and political philosopher Frantz Fanon in the seminal book ‘Black Skin, White Masks’ (1975). The findings are also contextualized within a critique of institutional racism, both historically and currently, and within an intersectional discussion of the need for structural competency and the provision of safety for racialized groups in clinical settings.

**Disclosure of Interest:**

None Declared

